# The Latest Research Progress on Bionic Artificial Hands: A Systematic Review

**DOI:** 10.3390/mi15070891

**Published:** 2024-07-08

**Authors:** Kai Guo, Jingxin Lu, Yuwen Wu, Xuhui Hu, Hongbo Yang

**Affiliations:** 1 Suzhou Institute of Biomedical Engineering and Technology, Chinese Academy of Sciences, Suzhou 215163, China; 2 School of Biomedical Engineering (Suzhou), Division of Life Sciences and Medicine, University of Science and Technology of China, Hefei 230026, China; 3 College of Mechanical and Electrical Engineering, Changchun University of Science and Technology, Changchun 130022, China

**Keywords:** prosthetic hand, transmission mode, control mode

## Abstract

Bionic prosthetic hands hold the potential to replicate the functionality of human hands. The use of bionic limbs can assist amputees in performing everyday activities. This article systematically reviews the research progress on bionic prostheses, with a focus on control mechanisms, sensory feedback integration, and mechanical design innovations. It emphasizes the use of bioelectrical signals, such as electromyography (EMG), for prosthetic control and discusses the application of machine learning algorithms to enhance the accuracy of gesture recognition. Additionally, the paper explores advancements in sensory feedback technologies, including tactile, visual, and auditory modalities, which enhance user interaction by providing essential environmental feedback. The mechanical design of prosthetic hands is also examined, with particular attention to achieving a balance between dexterity, weight, and durability. Our contribution consists of compiling current research trends and identifying key areas for future development, including the enhancement of control system integration and improving the aesthetic and functional resemblance of prostheses to natural limbs. This work aims to inform and inspire ongoing research that seeks to refine the utility and accessibility of prosthetic hands for amputees, emphasizing user-centric innovations.

## 1. Introduction

In June 2011, the World Health Organization published the highly anticipated World Report on Disability, which indicated that the global population of persons with disabilities had surpassed one billion, accounting for 15% of the world’s total population [1]. In China, statistics from the China Disabled Persons’ Federation as of the end of 2010 reported that the number of persons with disabilities exceeded 85.02 million, with 24.72 million suffering from limb disabilities, representing approximately 30% of the disabled population. In 2005, around 541,000 Americans experienced varying degrees of upper limb loss. Italy reports approximately 3500 cases of upper limb amputations annually, while the United Kingdom records about 5200 cases [2,3]. Trauma is the primary cause of upper limb amputations, followed by tumors and vascular or infectious diseases [3].

The human hand is a very precise and flexible device that plays a critical role in daily life, facilitating activities such as grasping, tactile perception, communication, and executing subtle and complex actions. The absence of a hand represents a significant physical trauma that can severely hinder an individual’s ability to perform daily tasks independently, potentially leading to unemployment and social isolation. While prosthetic limbs offer a way to restore some hand functions necessary for daily living and work, they are not always a mandatory solution, as some individuals may choose alternative adaptations. However, current prosthetic devices often lack the strength and flexibility found in natural hands. Most are capable of only limited, predefined gestures and do not fully replicate the nuanced movements of a human hand, highlighting a significant gap in design requirements. Additionally, despite advancements in prosthetic technology, the adoption rates of prosthetic hands remain low. Cost is a significant barrier, and the high-priced devices often do not deliver reliability or the ability to perform complex movements [4,5]. Well-performing anthropomorphic hands are invariably expensive, such as the BeBionic hand (Ottobock(Dudletown, Lower Saxony, Germany); approximately $11,000) and the i-Limb (Touch Bionics Inc. (Livingston, UK); approximately $18,000), prices which are unaffordable for most patients [6]. These economic factors severely limit accessibility and hinder the widespread use of advanced prosthetic technologies.

**Figure 1:**
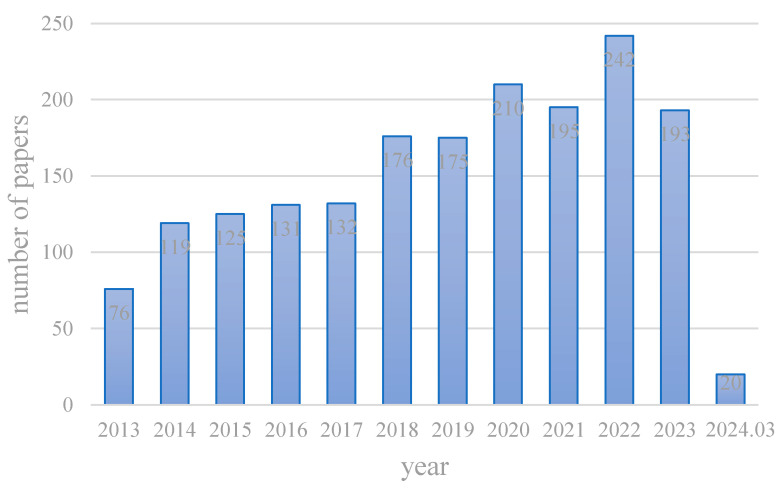
Trends in annual publications on prosthetic hand-related topics.

Despite advancements in science and technology, modern upper limb prostheses continue to face significant limitations. One of the main engineering challenges in developing these devices is the integration of actuators, sensors, and electronic components into a prosthesis that matches the size and weight of the replaced hand or limb. Furthermore, there is an urgent need to enhance control over these prostheses, as this significantly impacts their functionality [7]. Additionally, operating an artificial hand requires extensive training, and the lack of sensory feedback, along with the noise generated by actuators during movement, means these devices still do not fully meet user needs [5,8]. These challenges underscore the necessity for ongoing innovation and refinement in the design and functionality of prosthetic limbs to better meet the complex demands of their users [9]. As illustrated in Figure 1, the trend of annual publications on prosthetic hand-related topics in the Science database shows a growth pattern, indicating a promising future for the field of prosthetic hand research.

In the journal *Prosthetics and Orthotics International*, Fishman identified psychology as one of the six indispensable skills and knowledge areas in professional prosthetic and orthotic practice [10]. The appearance of a prosthesis is closely linked to a patient’s body image and social interactions. A prosthesis that more closely resembles a natural human limb can significantly enhance the user’s confidence in social settings and reduce negative emotions associated with limb loss. The demand for aesthetically pleasing prosthetics is not solely about functionality but also pertains to social interactions with others [10]. As technology advances, expectations for prosthetics extend beyond functional restoration to include aesthetic appeal. For instance, high-end bionic prostheses like the DEKA Arm and the BeBionic hand are not only technologically advanced but are also designed with an emphasis on realistic and aesthetically pleasing appearances [11,12]. Therefore, aesthetics is recognized as one of the essential aspects requiring improvement in prosthetic design. Numerous studies have harnessed human bioelectrical signals for the control of prosthetic hands, employing modalities such as the electroencephalogram (EEG), electrooculogram (EOG), EMG, and visual systems. Notably, the use of EMG signals to control muscles and electrical prostheses has shown considerable promise [13,14]. This approach enables amputees to manipulate robotic devices using the surface muscle signals detected on the residual forearm. The collection and analysis of bioelectrical signals generated during hand movements are crucial for recognizing hand movement intentions. It is essential to classify these muscle electrical signals into corresponding hand movement patterns through algorithmic analysis. In most published studies, researchers have utilized muscle electrical signals recorded during the gripping phase to categorize movement patterns, achieving satisfactory classification outcomes [15,16,17,18,19]. Thus, the development of controlled prosthetics through the identification of EMG signal patterns, including feature extraction and the application of machine learning-based classifiers, remains a primary research focus. The roles of EEG and EOG in the control of artificial limbs primarily support EMG-based control systems.

Individuals’ demands for equipment include idea identification, automatic control, anthropomorphic appearance, feedback, thumb performance, accuracy, heat dissipation, visual field operation, low noise, equipment use time, light weight, dexterity of fingers, stable grip, and other requirements [20,21,22,23]. Among them, feedback is of great help to help patients skillfully control their hands. The feedback information of prosthetic hands can be divided into sensory feedback information and motion feedback information. Sensory feedback mainly focuses on tactile, visual, auditory, and nerve stimulation, and it is realized by vibration, visual light, audio, and nerve stimulation [24]. Motion information feedback mainly includes position and force, and its detection methods mainly include current, voltage acquisition, and pressure acquisition. Realizing fine and dexterous control is a very complicated problem that needs to be solved, and it is still an active research field, because it not only needs to explain the intention of grasping control but also needs accurate feedback [25,26].

**Figure 2:**
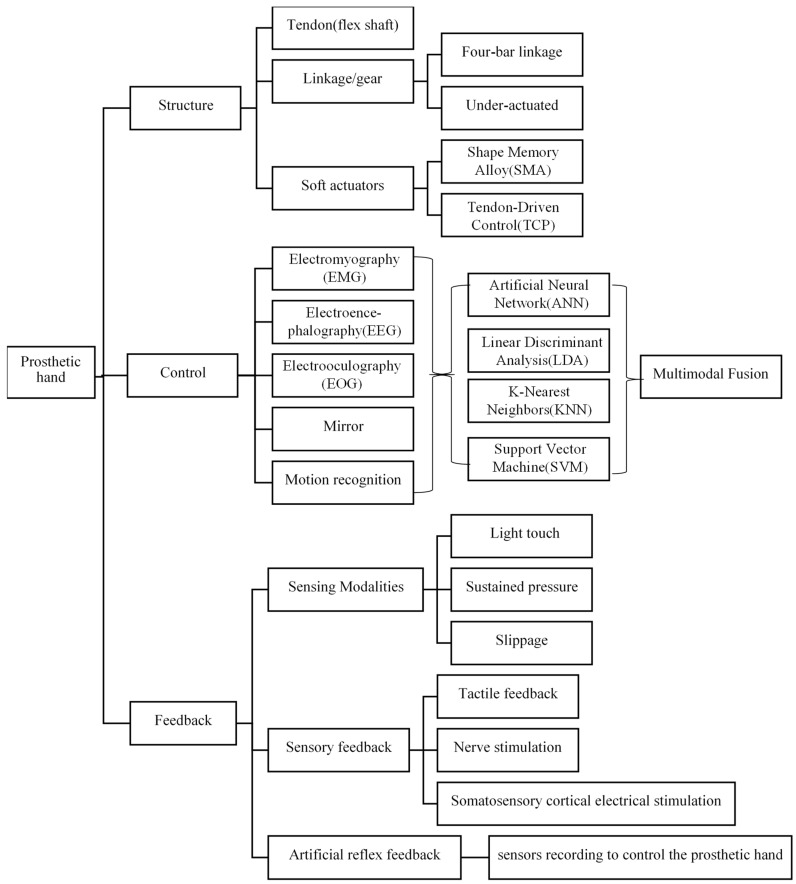
The basic outline of the contents covered by this article.

This article provides a detailed overview of the current advancements in prosthetic hand technology through extensive reviews of research from institutions, commercial product analyses, and scholarly articles. In the abstract, we expand on several aspects, systematically evaluating the progress in prosthetic hand development and detailing the classifications, comparisons, and designs of transmission mechanisms, control strategies, dimensions, and feedback techniques. Figure 2 presents a basic schematic of the topics covered in this article. The subsequent organization of this article is structured as follows: The paper begins by examining the intricacies of the human hand, covering metacarpal, finger, and thumb models. It then delves into the characteristics of prosthetic hands, discussing their size, weight, and commercial variations. Following this, various structural designs of prosthetic hands are analyzed. Control and function aspects are explored next, including main control methods, feedback mechanisms, and a functional comparison. Lastly, the discussion and conclusions sections synthesize findings and outline future implications.

## 2. Characteristics of the Human Hand

The hand represents the most critical component of the human upper limb kinematic structure. It consists of 27 bones, 21 degrees of freedom, and 37 muscles [27]. Specifically, the four fingers each consist of a metacarpophalangeal (MCP) joint, a proximal interphalangeal (PIP) joint, and a distal interphalangeal (DIP) joint, while the thumb features a carpometacarpal (CMC) joint, an MCP joint, and an interphalangeal (IP) joint [28]. This coordination between muscles, bones, and joints facilitates a wide range of precise movements in the hand. This intricate assembly allows for significant dexterity and mobility within the fingers. Many prosthetic hands seek to emulate this structure to replicate similar functionality.

### 2.1. Metacarpal Model

The skeletal structure of the palm is formed by the metacarpal and carpal bones. The last four carpometacarpal joints facilitate a limited range of flexion–extension movements between the carpals and metacarpals, with the range incrementally increasing from the index to the little finger. The CMC joint of the little finger is unique, being a saddle joint with an oblique axis, which allows for coupled rotation and adduction when flexed, resulting in a cupped palm shape [29].

### 2.2. Finger Models

Each finger consists of three phalanges: proximal, intermediate, and distal. The proximal and distal interphalangeal joints each have a single flexion–extension axis. The metacarpophalangeal joints, located at the base of the fingers, feature axes for both flexion–extension and abduction–adduction.

#### 2.2.1. Metacarpophalangeal Joints

MCP joints, endowed with two degrees of freedom, facilitate both flexion–extension and abduction–adduction movements. The active flexion typically reaches about 90°, extension ranges from 30 to 40°, and some individuals may achieve up to 90° in passive extension. The abduction–adduction movement spans approximately 30° [28]. Furthermore, these joints exhibit minimal passive axial rotation. Generally, MCP joints are modeled as universal joints with orthogonal, intersecting axes [30]. Kinematically, substantial axial rotation coupled with abduction–adduction is observed in the proximal phalanx when the finger flexes, while extensive abduction–adduction with minimal axial rotation occurs upon extension.

#### 2.2.2. Proximal and Distal Interphalangeal Joints

The PIP and DIP joints function as one-dimensional hinges linking the three phalanges, with the PIP flexing over 90° and the DIP slightly less. Active extension in these joints is negligible, although the DIP can exhibit up to 30° in passive extension. These joints also show a minor passive abduction–adduction movement, particularly the DIP. The orientation of these joint axes, initially perpendicular in full extension, progressively tilts during flexion. The alignment of the MCP, PIP, and DIP axes is non-parallel. Despite these variations, it is commonly assumed that the finger kinematic chains are planar, employing a single fixed axis for each joint [29,30].

### 2.3. Thumb Models

The thumb includes three joints: the trapeziometacarpal (TM) or carpometacarpal (CMC), the metacarpophalangeal (MCP), and the interphalangeal (IP). Modeling the thumb is complex due to the TM and MCP joints, each having two degrees of freedom and being capable of movement across all three anatomical planes.

#### 2.3.1. Trapeziometacarpal Joint

The TM joint possesses dual axes that are non-intersecting and non-orthogonal. The MCP axes are challenging to define as they invariably coordinate with TM movements due to shared musculature [29]. Consequently, simulating healthy thumb movement may require fewer than four degrees of freedom for the combined TM and MCP motion.

#### 2.3.2. Interphalangeal Joint

The thumb’s IP joint is widely recognized as best modeled with a single degree of freedom [29]. Its axis, parallel to the flexion creases, is situated at 90 ± 5% of the proximal phalanx’s length, oriented at an angle of 83 ± 4° relative to the midline and 5 ± 2° to the palmar surface of the bone.

## 3. Characteristics of Prosthetic Hands

### 3.1. The Size and Weight of the Prosthetic Hand

The design of prosthetic hands must prioritize lightness, as the comfort of the prosthesis is directly linked to its weight. Typically, the weight of human hands accounts for approximately 0.5% and 0.6% of total body weight for men and women, respectively, with the average weight of a man’s hand being around 426 g. Designing a prosthetic hand that closely mimics this natural weight is a significant challenge. Lightness in prosthetics is crucial because excessive weight can lead to physical discomfort and pain, prompting some users to abandon their use. Commercially available prosthetic hands generally weigh about 500 g, with much of this weight attributable to their complex structures. The weight contribution of each component, particularly the fingers, is influenced by several factors, including the number of fingers, the design of the mechanism, the geometric dimensions, and the materials used.

The compactness of a structure directly correlates with the reduction in the weight of prosthetic fingers. Utilizing tendons, SMA, thermally controlled polymer muscles (TCPM), and other transmission mechanisms can significantly contribute to the lightweight design of prosthetic hands [31]. Table 1 presents relevant data on the degrees of freedom, structural dimensions, weight, and output force of prosthetic hands developed in recent years. Notably, ‘FF’ refers to fingertip functionality and ‘PG’ denotes grasping power. The data illustrate a clear trend in prosthetic hand design towards increased degrees of freedom, reduced volume, decreased weight, and enhanced output force [32].

**Figure 3:**
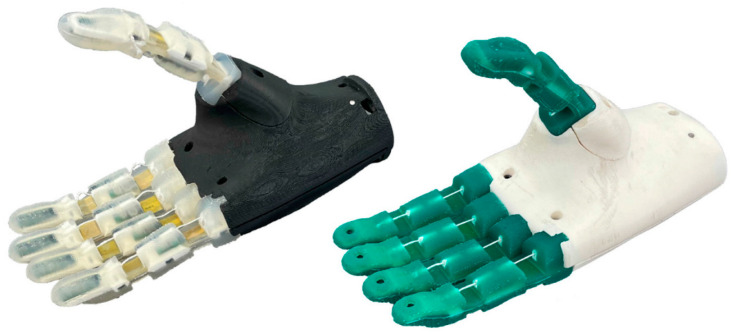
KIT prosthetic hand [38].

Among the innovations, the intelligent electromechanical integrated prosthesis developed by Pascal Weiner and colleagues stands out. This advanced prosthesis features adaptive drivers, multimodal sensing, and airborne computing resources that facilitate autonomous and intuitive control. As depicted in Figure 3, the prosthetic models are differentiated by male and female designs, with scalable hand sizes to accommodate various user needs. These prosthetics utilize an underactuated mechanism, allowing them to conform to the shape of any object they grasp. Additionally, each hand incorporates an embedded system within the palm for processing sensory data and controlling the device, enhancing the user’s manipulation capabilities.

**Figure 4:**
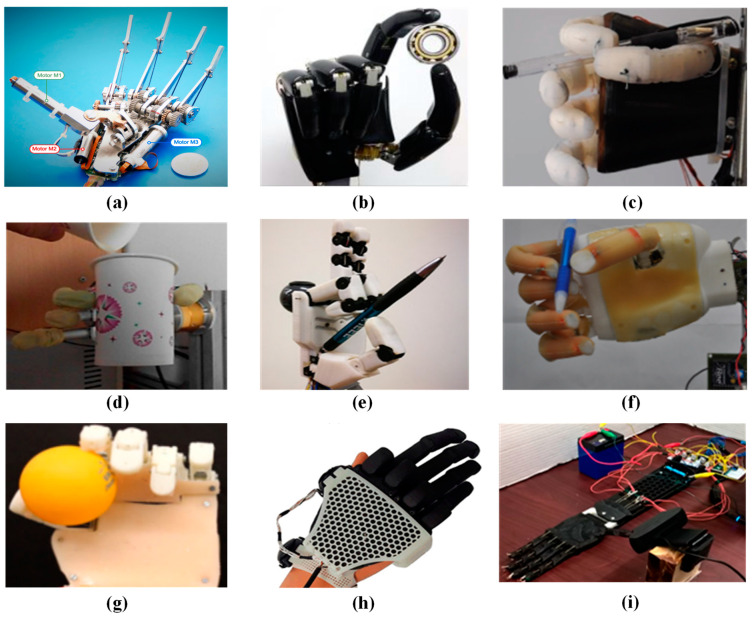
Physical image of the rest of the hand. (**a**) SSSA-MyHand [33]; (**b**) Jeong et al. [34]; (**c**) SoftBionic hand [35]; (**d**) Zhang et al. [36]; (**e**) Galileo hand [37]; (**f**) UC Softhand [39]; (**g**) TCP UTD hand [40]; (**h**) Developed prosthetic hand [41]; (**i**) Coiled SMA [42].

Figure 4 provides a physical representation of these prosthetic hands, illustrating the practical application of the described technologies. The data concerning the weight and size of the prosthetic hands listed in Table 1 are derived from published studies. Gathering comprehensive data on the total weight of the system, including the connecting cavity between the prosthetic and the human body, the impact of energy consumption on battery size, and the weight of the primary equipment, presents challenges. Nonetheless, it is evident that the weight requirements for these prosthetics are stringent. To maximize user comfort and the device’s load-bearing capacity, minimizing the weight of the prosthetic is imperative.

### 3.2. Characteristics of the Commercial Prosthetic Hand

**Figure 5:**
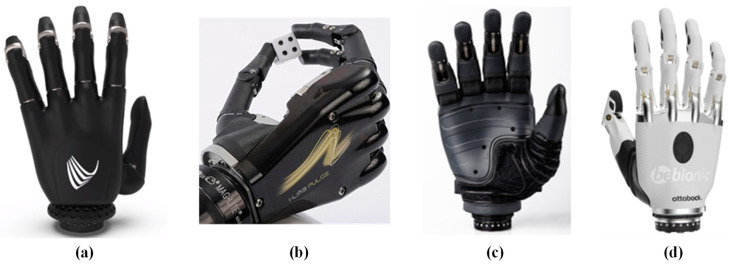
Commercial prosthetic hand. (**a**) Vincent Hand [44]; (**b**) i-Limb [3,45]; (**c**) Taska Hand [8,20]; (**d**) Bebionic Hand [3,7].

Commercial prosthetic hands are predominantly controlled by EMG, a method that harnesses the residual bioelectrical signals from the arm to dictate the movement of fingers. This control is achieved through the strategic placement of electrodes along the residual limb, which capture the electrical activity generated by muscle contractions. These signals are then amplified and processed to drive the movement of each finger individually, typically facilitated by a single motor per finger within a connecting rod structure. This configuration not only ensures the reliability of movements but also enables significant grasping force, as illustrated in Figure 5.

In detail, the EMG control mechanism involves the use of surface electrodes that are non-invasively attached to the skin over the muscles. The distribution of these electrodes is crucial for accurate signal acquisition and typically involves multiple channels to capture a comprehensive range of muscle activity. Modern bionic hands often employ between 8 and 16 channels, depending on the complexity of the desired movements and the control system’s sophistication.

The precision of EMG signal collection is influenced by several factors, including the placement of electrodes, the quality of the electrode–skin contact, and the signal processing algorithms used. Advanced filtering and signal processing techniques are essential to minimize noise and enhance the clarity of the bioelectrical signals. This precise collection and interpretation of EMG data allow for more nuanced and controlled movements of the prosthetic fingers, enabling users to perform a variety of tasks with greater efficacy.

Additionally, while EMG is the primary method for controlling commercial prosthetic hands, other physiological signals such as electroencephalography (EEG) are also explored in research contexts for their potential to provide more direct brain-controlled functionalities. Like EMG, EEG involves the collection of electrical signals, but it captures brain activity rather than muscle activity, typically requiring a different setup and more sophisticated signal processing techniques.

Through innovation in materials and technology, the service life is prolonged, while the burden on the hand is reduced, and more possibilities are given to the prosthesis. For example, the index finger of the prosthetic hand as shown in Figure 5a can be used on a touch screen, and the prosthetic hand in Figure 5b,d can operate the prosthesis through Bluetooth connection. The damping knuckle block designed in Figure 5c provides a high level of impact protection for the prosthesis. The controller built into the prosthetic hand listed here can directly connect the EMG sensor to the arm. Users can choose a self-defined motion control mode through functions, so as to easily and accurately control the prosthesis. The weight range of prosthetic hands is 400–600 g, and the output force is 60 N–140 N. The common goal pursued by the design is to move towards lighter weight, and better dexterity and personification. In addition, the waterproofness of prosthetic hands has become one of the considerations of commercial design.

The market features a range of highly acclaimed prosthetic models, each tailored with unique advantages and designed to address specific user requirements. The Vincent Hand, known for its natural grip pattern and durable design, offers multiple grip settings to cater to varied tasks. Its most notable benefit is cost-effectiveness relative to other advanced prosthetics. However, its limited sensory feedback could potentially compromise user dexterity, and it offers fewer aesthetic customization options. Primarily, this model is suited for everyday activities, particularly for users who need robust and reliable support without advanced functionalities.

The i-Limb stands out with its individually powered digits, enabling highly complex movements. This prosthetic is further enhanced by a mobile application that facilitates easy adjustments and customization of grip patterns. It also boasts a superior aesthetic design that closely mimics a natural hand’s appearance. Nonetheless, these advanced features come at a higher cost, and the prosthetic requires regular maintenance and software updates, making it ideal for environments where high dexterity is essential.

Taska Hand is distinguished as the first waterproof prosthetic hand, designed for rigorous use, including activities in wet conditions. It features a robust design with tactile feedback capabilities, offering substantial durability. However, its heavier weight may lead to user fatigue, and the high cost associated with its advanced features makes it most suitable for active users, particularly those involved in physical labor or outdoor activities where durability is crucial under challenging conditions.

The Bebionic Hand offers extensive grip patterns and control options, enabled by advanced sensors. It is highly customizable in both fit and aesthetics, including options for skin tone covers, and is renowned for its strong and precise grip. However, the complexity of this hand introduces a learning curve for new users, and its significant power consumption requires frequent recharging. This model is ideally suited for daily use and professional tasks that demand precise manual dexterity.

Each of these prosthetic hands is designed with specific target users in mind, ensuring that they not only meet but exceed the functional requirements expected in various real-world applications.

## 4. Different Structural Designs of Prosthetic Hands

To closely mimic the functionality of human hands, researchers have designed numerous intelligent anthropomorphic prosthetic structures. Many effective prosthetic hands with high adaptability or low degrees of freedom have been developed for efficient grasping exercises. The representative core transmission mechanisms of these prosthetic hands are divided into tendon drive, linkage drive, and direct motor drive structures [45]. Table 2 lists the characteristics of these different transmission methods and their respective advantages and disadvantages.

In the direct motor drive structure, each finger is equipped with an independent small motor that directly controls the movement of the finger, providing precise and independent finger control. This design allows each finger to perform independent actions, significantly increasing the hand’s flexibility and usability. The advantage of the direct motor drive structure is its ability to perform complex finger movements, such as fine grasping and independent coordination between fingers. However, the drawbacks include higher energy consumption and increased weight, as a motor and corresponding control circuits are required for each finger.

**Figure 6:**
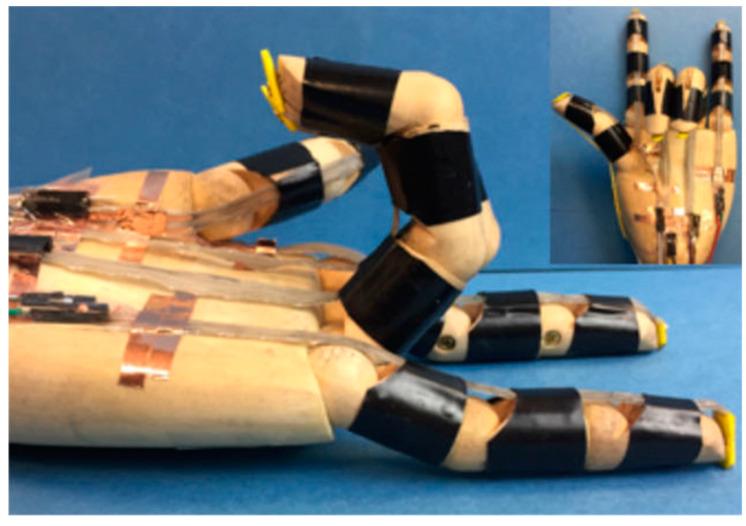
Artificial thermal deformation tendon prosthetic hand [46].

As shown in Figure 6, artificial tendons drive the finger movements of the bionic hand, utilizing a combination of liquid crystal elastomers (LCE) and liquid metal heating elements. Heating causes the tendon’s temperature to rise, leading to linear contraction and thus enabling the bionic prosthetic fingers to bend in a human-like manner. This tendon-driven approach mimics the natural motion of human fingers but may have limitations in precise control and rapid response compared to the direct motor drive.

**Figure 7:**
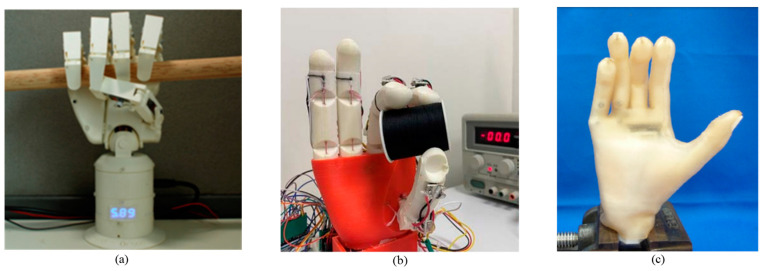
Combined structured prosthetic hand. (**a**) MCR-Hand II [47]; (**b**) Integration of Soft Bend Sensor Chambers in Prosthetic Hand Joints for Enhanced Gesture Control [48]; (**c**) Comparison of Ultra-Elastic Rubber Gloves with PVC in Myoelectric Prosthetic Hands for Realism and Flexibility [49].

In Figure 7, there are also some combined structures, such as Yang Hansen combined with the advantages of tendon drivers and connecting rod drivers, and designed as a compact mechanical structure to imitate the flexibility of human hands [47]. Based on studying the manual anatomy structure and the existing related robotics, the MCR-Hand II mechanical design was proposed to develop the prototype of the robot. As shown in Figure 7a, it integrates the underlying control system and experimental research. The experimental results show that the robot can achieve the capture and operation of most objects.

Z Hao et al. developed a 3D-printed soft prosthetic hand with an embedded soft sensor [48], which is designed for the use of prosthetic limbs to effectively operate with the new generation of muscle-powered control systems. As shown in Figure 7b, a soft bend sensor chamber is embedded in each joint by adding a soft position sensor to each joint. Users can monitor the position of their fingers to avoid self-collision. The combination of different gestures allows prosthetic hands to perform multiple stages to grab and carry multiple objects at the same time. Yabuki Yoshiko used two different ultra-elastic rubber–thermoplastic styrene elastomers (TSE) and silicon rubber to make new makeup gloves [49]. As shown in Figure 7c, they were compared with the polyvinyl chloride (PVC) gloves used in the myoelectric prosthetic hands, achieving realistic appearance and flexible movement.

Currently, topology optimization methods have become a popular approach in the structural design of biomimetic underactuated artificial fingers or hands. Y Zheng et al. [50] proposed a novel finger design tailored for prosthetic applications, employing a family-based topology optimization method for structural synthesis and dimensional analysis. The results indicate that the flexible prosthetic fingers meet the design requirements and address some issues inherent in traditional prosthetic fingers. Meanwhile, Y Sun et al. [51] developed a dual-finger gripper based on continuous structures for adaptive grasping. They utilized a 3D topology optimization design approach, incorporating additional springs into the design problem to facilitate the adaptive adjustment of mechanical fingers, enabling successful grasping of objects with varying shapes and materials.

**Table 2:** Transmission structure characteristics of prosthetic hands in recent years.

Structure	Paper	Author (year)	Features
Tendon-Driven	[52]	Elif Hocaoglu et al. 2022	Functionality: Tendon-driven systems mimic the natural movement of human hands by using synthetic tendons that pull on the fingers, allowing them to flex and extend. This system closely replicates the fine motor movements of biological hands. Design: The tendons are typically made from durable materials such as nylon or other high-tensile fibers that offer both strength and flexibility. These tendons are connected to motors or actuators that control their movement. Advantage: The prosthetic hand based on the tendon drive mechanism is most similar to the human hand, which can achieve almost similar movement to the human hand and generate high fingertip force according to the tendon connection configuration. Easy to implement the lightweight design. Disadvantage: The assembly and maintenance of prosthetic limbs increase costs and complicate repairs, especially with tendon issues. If a tendon is damaged, its complex structure makes repairs challenging. When two tendons are attached to a joint, pre-tensioning is recommended to manage friction and efficiency. Also, installing a return spring is necessary when a single tendon is connected, though it complicates force control and affects the hand’s responsiveness. Notably, synthetic muscles like Liquid Crystal Elastomers show inherent elasticity, influencing design considerations and potentially reducing the need for additional components.
	[53]	Maria Claudia F. Castro et al. 2022	
	[46]	Haiqing Lu et al. 2021	
	[54]	Geng Gao et al. 2021	
	[55]	Danilo Estay et al. 2021	
	[56]	Larisa Dunai et al. 2021	
	[57]	Te-Ru Chen et al. 2021	
	[58]	Hao Zhou et al. 2019	
	[59]	Inhoe Ku et al. 2019	
	[60]	Furui, Akira et al. 2019	
	[61]	Cosimo Della Santina et al. 2018	
	[62]	M. Laffranchi et al. 2020	
	[63]	Fonseca, G. 2023	
	[64]	Yong, X. 2023	
Linkage-Driven	[65]	Yanchao Wang et al. 2022	Functionality: Linkage-driven systems use mechanical linkages to transfer motion and force from the motors to the fingers. These systems often involve a series of rigid links connected by pivots or joints. Design: The linkages are typically made from metals or durable polymers designed to withstand the mechanical stresses during hand operations. The design of the linkage determines the motion path of the prosthetic fingers. Advantage: Strong, easy to manufacture and maintain, high output force, strong stability. Disadvantage: The connecting rod is relatively thick and hard, so it is difficult to achieve multi-freedom exercise and maintain a larger working space.
	[66]	Jacob L. Segil et al. 2021	
	[67]	Zachary Yoder et al. 2020	
	[68]	Mohamad Aizat Abdul Wahit 2020	
	[69]	Wooseok Ryu et al. 2020	
	[70]	Kyung Yun Choi 2017	
Motor-Driven	[71]	Dong-Hyuk Lee et al. 2017	Functionality: Motor-driven systems directly incorporate small motors in each finger, allowing individual control of finger movements. This design provides a high degree of autonomy and precision. Design: These systems often use micro-motors housed within the fingers or palm of the prosthetic hand. The integration of motors with electronic controllers facilitates complex and varied finger movements. Advantage: Finger joint positioning motors drive the joint directly or using pulleys. This structure has high joint-driving efficiency, and it is easy to arrange the joints in the expected position. Disadvantage: The size and performance of prosthetic hands depend on motors, and high-quality motors increase costs. Because the weight of the motor can cause large inertia, complicated control is needed. The space between the fingers is very narrow, and it is difficult to connect the force sensor to the finger. It is difficult to achieve compactness and high performance.
	[43]	Vanich, P. 2023	

According to the analysis of Table 2, to design a manipulator with high dexterity and multiple degrees of freedom, the positioning structure of the finger joint should be composed of a connecting rod and a rotating shaft, and the rope, as the driving structure, is designed to provide driving force outside the finger. The flexion, extension, and swing of fingers are controlled by the movement of the upper, lower, left, and right ropes, which can not only ensure the accuracy of the movement position of fingers but also transfer the weight of the equipment to the far end through the rope drive, thus realizing lightweight design and high output force.

## 5. Control and Function of Prosthetic Hands

### 5.1. Main Control and Feedback Methods of Prosthetic Hands

To realize the control of intelligent prosthetic limbs, collecting and analyzing the residual information of upper limbs is the first step in identifying the movement intention. The sources of information, including human sports intentions, include biological electrical signals, motion signals, and voice signals. The biological electrical signals mainly include EMG signals, EEG signals, and EOG signals [72]. Muscle electrical control prosthesis is one of the most widely used prosthetic limbs at present [50,51,73,74]. Although the relevant gesture recognition system based on brain electrical, voice signals, and intentional recognition has been established, in addition to muscle electrical control, there are currently few other control methods that can be used in intelligent control of prosthetic limbs alone [75,76,77,78,79,80]. Other control methods are fusion or feedback with muscle power control methods to increase the intelligent control of prosthetic hands. Table 3 shows some of the control methods, feedback information, and characteristics of some smart prosthetic hands in recent years.

It can be seen in Table 3 that muscle electricity prosthesis has the most widely used application. Prosthetic hand control is primarily divided into two major categories: direct control and pattern recognition control. Direct control leverages the user’s residual neuromuscular activity, such as EMG signals, to command the prosthetic limb’s movements directly. Conversely, pattern recognition control utilizes machine learning algorithms to classify diverse muscle movement patterns, thereby managing the prosthetic’s functional output. Feedback mechanisms are integral to the user for effective operation of prosthetic hands, generally categorized into sensory feedback and visual feedback. Sensory feedback typically involves mechanical vibration and electrical stimulation to emulate touch and pressure sensations. Visual feedback, on the other hand, often relies on external devices that provide visual cues regarding the prosthetic’s position and its interaction with environmental objects. In the realm of prosthetic hand control, musculoskeletal models play a crucial role by simulating the physical dynamics and biomechanics of human hand movements. With the advancement of artificial intelligence, machine learning algorithms have become pivotal in recognizing and categorizing muscle activity patterns to predict and facilitate hand movements. Additionally, deep learning methods are employed to process a wider array of inputs, enhancing the accuracy of the prosthetic’s movements, thus enabling more precise and controlled operations.

**Figure 8:**
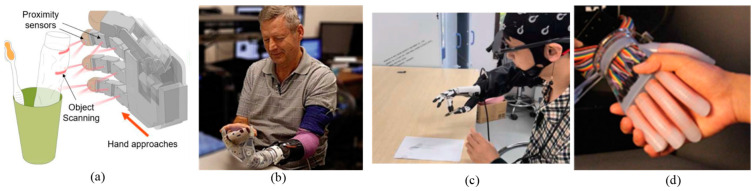
New control method of a prosthetic hand: (**a**) Seamless controls for dexterous prosthetic hands [102]; (**b**) Improving the dexterity of a bionic hand by biomimetic sensory feedback of peripheral nerve stimulation [103]; (**c**) AR-SSVEP enhancement, asynchronous control, and machine vision-assisted method of brain control of a prosthetic hand [104]; (**d**) Based on tensile optical waveguide photoelectric neural soft rubber [105].

In addition, there are some other control methods and different information feedback methods [95,96,97,98,99,100]. For example, Moaed A. Abd et al. developed a new type of multi-channel wearable soft robot arm belt to convey artificial touch to prosthetic hands [101]. As shown in Figure 8a, Si-Hwan Heo et al. proposed a novel perception system that provides grasping intelligence to the prosthetic hand. The proximity perception-based grasping intelligence (P2GI) system comprises a proximity sensor system and a prompt decision-making process. The proximity sensors embedded in the prosthetic hand map the point cloud of the object in real time while the prosthetic hand reaches toward the object. Simultaneously, a real-time decision-making algorithm infers the user’s intended grasp posture by obtaining the hand–object relation from the point cloud data. The finger motion that stably grasps the target object with the inferred grasp posture is planned accordingly [102].

J. A. George et al. describes a two-way neuromuscular prosthetic hand, using bionic sensation feedback. As shown in Figure 8b, through the activation of the contact sensor on the prosthesis, the long-term implanted electrode array can stimulate the residual sensory nerve fibers in the nerve, thus arousing tactile sensation on the affected limb. With the role of sensory feedback, participants show a more accurate grip and can better deal with fragile objects [103]. Figure 8c is the research of Zhang and Xiaodong. It aims to propose an asynchronous pattern recognition algorithm by integrating augmented reality technology, an innovative visual stimulus paradigm, and an asynchronous decoding/control strategy, so as to improve the interactive logic and practical application of the prosthetic hand and BCI system [104].

Huichan Zhao et al. proposed the application of a stretchable optical waveguide in strain sensing of a prosthetic hand [105]. These optoelectronic strain sensors are easy to fabricate and demonstrate low hysteresis and high precision in their output signals. As shown in Figure 8d, as a demonstration of their potential, the photonic strain sensors were used as curvature, elongation, and force sensors integrated into a fiber-reinforced soft prosthetic hand.

In addition, many of the problems associated with signals from surface electromyography can be overcome with invasive electromyography technology, which uses sensors implanted into muscle, or over the surface of muscle yet below the subcutaneous layer [106,107,108]. For example, Ripple Neuro has been developing an implantable neural technology device, which can record electromyography and low-density meter signals without the need of implanting batteries or percutaneous leads. The feedback implant system developed by Dustin Tyler et al. uses the electrical stimulation of implanted peripheral nerve cuff electrodes to produce touch perceptions in many positions of the affected limb of the human subject, which enables human subjects to better manipulate delicate objects [109,110]. IMES, of the Alfred E. Mann Scientific Research Foundation, a medical implant device inserted into human tissues through surgery, is used to detect the electrical signals of human muscles and act on them [111,112]. The IMES implant has been stable for more than 4 years without excessive rejection [113], and the long-term implanted muscle membrane electrode can achieve high-quality direct control within several months of surgery [114]. These applications made the implantable signal acquisition device become a reality in clinical application.

### 5.2. Functional Comparison of Prosthetic Hands

In the research of the prosthetic hand, a machine learning algorithm is used to control the motion mode of the hand, and the algorithm is the main condition to determine the accuracy of prosthetic limb intelligent motion [115,116]. Table 4 shows a comparative analysis of the control techniques and effects of artificial hands.

For example, R Zhang et al. [117] studied the statistical analysis of EMG values calculated from surface EMG signals recorded from six muscles of 20 healthy subjects in 2022. The results of the statistical analysis indicate differences between right-hand sEMG signals recorded from females and males. In addition, two types of machine learning algorithms for hand gesture recognition that considered sex differences were proposed based on sex differences in sEMG signals of upper limb muscles. These two methods were applied to KNN (k-nearest neighbor), SVM (support vector machine), and ANN (artificial neural network) classifiers, demonstrating that taking sex differences into account can improve the accuracy of hand movement recognition predictions. Compared with the other two classification algorithms, the ANN classification algorithm with gender labels has the best classification performance, with an average prediction accuracy of 98.4%, while the KNN and SVM classifier, with the gender label method, have prediction accuracies of 94% and 94.2%, respectively [117,118]. The biggest difference between KNN and SVM is that all training sample points are used in KNN prediction, which is a non-sparse model; SVM only uses a support vector, a sparse model.

X Li et al. proposed an identification method based on force muscle diagrams; the robustness of the proposed method was investigated, and the classification accuracies were above 90% even when the white noise ratio increased to 50%. This study demonstrated the effectiveness of the RSC-based pattern recognition method for motion classification [119].

M Coskun et al. proposed a one-dimensional convolution neural network model using surface EMG signals, which achieved the highest accuracy rate of 94.94% when recognizing and classifying six gestures in 2020 [120].

Z Zhang et al. identified 11 finger movements through time domain feature extraction and an artificial neural network in 2020. The accuracy of identification reached 91.10%. Then, he continuously eliminated the channels that contributed the least to the recognition accuracy to find the best relationship between the number of channels and the recognition accuracy. Finally, it was found that the average recognition accuracy of seven channels was 90.52% [121].

A Altameem et al. used different feature extraction techniques and sophisticated machine learning algorithms to classify hand movements from EEG brain signals to control prosthetic hands for amputees in 2022. To achieve good classification accuracy, EEG signal denoising and feature extraction are very important steps [122].

G Pan et al. proposed to use recurrent neural networks (RNNs) to exploit the temporal information in ECoG signals for hand gesture decoding in 2018, decoded three hand gestures using the ECoG signals of two participants, and achieved an accuracy of 90%. They also studied the possibility of recognizing gestures within the shortest possible time interval after the start of exercise [123].

Ghazaei, G et al., based on deep learning artificial visual systems in 2017, classified the object based on the grip mode and trained the convolutional neural network with grabbing image training. After improving the network camera, the overall success rate of the subject’s operating target objects was as high as 80% [124].

To sum up, it can be seen that with continuous research on the control method of a prosthetic limb, the accuracy of the control of the prosthetic limb has been improved [125,126]. Whether it is EMG, EEG, artificial vision control, or gesture recognition, the accuracy rate is above 80%. Hand motion recognition controlled by EMG is also optimized by the algorithm, and the accuracy rate is as high as 98.4%, which provides a bright future for the prosthetic.

## 6. Discussion

The prostheses reviewed in this article demonstrate that designers have consistently prioritized user needs, focusing on anthropomorphic attributes such as weight, size, structure, and control. Recently, gesture recognition technologies based on human biological signal recognition have advanced significantly. However, their application remains largely confined to experimental and laboratory settings and has not yet achieved widespread commercial use or integration into everyday user environments [127,128,129,130]. The intersection of scientific research and commercial interests in this cutting-edge field reveals differing starting points. While the business community prioritizes the actual needs of users with a focus on clinical applicability, academic institutions often explore broader research questions that may not yet address immediate practical applications. Reviewing the progress in prosthetic hands and research driven by human biological signal control highlights continuous innovation but also underscores persistent challenges that merit further exploration [131,132,133,134,135].

Firstly, structural design needs ongoing improvement to achieve lighter weight, higher degrees of freedom, more flexible transmission systems, and simpler control mechanisms. Secondly, significant issues remain in harnessing human biological signals for intelligent control. Currently, intelligent motion control of prosthetic hands primarily involves the collection and data processing of signals such as EMG, EOG, and EEG, demanding enhanced techniques for signal data acquisition, storage, and analysis.

The use of electromyography (EMG) is predominant in both research and practical applications, where prosthetic hands are controlled to perform basic actions like grasping and finger movements. While substantial progress has been made in recognizing upper limb motion patterns through surface muscle signals in controlled environments, the translation of these successes to real-time application is limited. Surface muscle signals are inherently non-stationary; their statistical properties change over time and are influenced by individual differences, external factors, and physiological changes, such as muscle fatigue. These variations can lead to mode misclassification and impact the accuracy of the system. Moreover, muscle–electric prostheses often face limitations in practical use, especially among young patients who experience ongoing physiological changes. The low spatial resolution and adaptability issues of these devices frequently lead to abandonment. This underscores the need for developing more universal biological signal controls for prosthetic hands that transcend the limitations of EMG.

**Figure 9:**
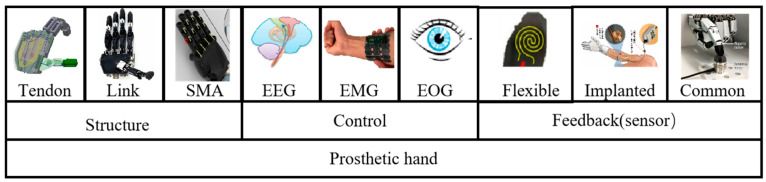
Concept map of prosthesis research direction.

A typical control flow based on vision involves several steps: capturing the scene, collecting EMG signals, extracting necessary information from the image, selecting a grasping posture, and using the EMG signal to initiate the grasp. This method requires significant computational resources, especially with recent attempts to apply deep CNN models to input images, further increasing the computational burden. Figure 9 shows the concept diagram of the research direction of prosthetic hands. Future work should include a few of these development directions as a way to solve the specific needs and desires of user control equipment.

Furthermore, sEMG pattern recognition continues to face challenges in clinical and home settings due to signal variability caused by electrode displacement and changes in skin condition. Implantable EMG sensors, which offer more stable and high-fidelity signals, may provide a viable solution to these issues, suggesting that EMG could remain a predominant signal for prosthetic hand control.

## 7. Conclusions

This review summarizes existing prosthetic hand technology using a systematic approach. Specifically, we investigated the development, structure, control modes, and weight characteristics of prosthetic hands. This paper proposes methodologies needed to design a multi-degree-of-freedom prosthetic hand. In terms of control methods, compared to EEG and EOG, EMG is more accurate and capable of recognizing a greater diversity of gestures. In theory, EEG can also be used to control prosthetic hands. Implantable selective cortical electrodes can record signals from hundreds of cortical neurons to control prosthetic hands. This invasive brain interface has potential, but is limited by the necessity for brain surgery (which most amputees are unlikely to accept) and limited functionality (regarding peripheral interfaces). Non-invasive brain interfaces can be applied on a large scale, but do not provide the performance control level usually required. EOG allows users to select the grasping mode of the prosthetic hand through eye movements, and ultimately control the opening and closing movements of the prosthetic hand through EMG signals, enhancing the timeliness and flexibility of the prosthetic hand, though it is difficult to use alone.

Looking ahead, the development of prosthetic hands will not only benefit from improved shared control between the prosthetic and the patient but more importantly, from the ability to fuse multi-modal information data to infer human intentions. By adjusting the weights of control factors such as electromyography, electroencephalography, vision, and mirror control in different scenarios, the control effect on the prosthetic hand will be enhanced. This will assist in the motion planning and control of the prosthetic hand, thereby improving its overall performance.

Additionally, with the rapid development of machine learning and artificial intelligence technologies, applying these technologies to the control systems of prosthetic hands can greatly enhance their adaptability and responsiveness. For example, using machine learning algorithms to optimize the perception and control strategies of the prosthetic hand can adjust and refine the hand’s motion responses in real time, making them more natural and suited to the user’s habits. Future research could explore how to integrate these advanced algorithms more effectively to achieve more precise and personalized prosthetic control. These technological innovations not only promise to improve the functionality of prosthetics but also help enhance the quality of life and self-efficacy of users.

## Figures and Tables

**Table 1:** Basic characteristic parameters of a prosthetic hand.

Prosthesis	Dof	Size/mm	Weight/g	Force/N
SSSA-MyHand [33]	10	200 × 84 × 56	478	9.4–14.6 FF
Jeong et al. [34]	11		380	15.7–48.2 FF
SoftBionic hand [35]	10	200 × 91 × 40	285	
Zhang et al. [36]	11	171 × 80.2 × 27.4	450	8–12 FF
Galileo hand [37]	15	162 × 69.6 × 25	350	50 PG
KIT Prosthetic hand [38]	10	194 × 77 × 28	377	9.0–12.3 FF
UC Softhand [39]	10	230 × 100 × 40	280	4.7–7.3 PG
TCP UTD hand [40]	16	122 × 64 × 14	140	0.4 FF
Developed prosthetic hand [41]	18	174 × 77 × 11	152.32	6.5–7.5 PG
Coiled shape memory alloys (SMA) [42]	12	425 × 120 × 75	656	1.1 PG
Single-DoF Prosthetic Hand [43]	1		540	80.2 PG

**Table 3:** Methods and features of prosthetic hand control in recent years.

Control	Paper	Author (Year)	Feedback	Features
sEMG	[81]	Daniel Johansen (2021)		Mixed tongue muscle control
	[82]	Federica Barontini (2021)	Tactile/vibration	Pneumatic feedback
	[83]	Moaed A. Abd (2021)	Tactile	The liquid metal tactile sensor
	[84]	Pascal Weiner (2020)	Tactile	Combination of the multi-mode sensor system
	[85]	Alireza Mohammadi (2020)		Three types of grip
	[86]	Joseph DeGol (2020)	Vision	Automatically grab selection
	[87]	Dimitra Blana (2020)	Anthology signal	Use the biomechanics of the limbs to explain the remaining muscle signals
	[88]	Patel, G. K. (2016)	Tactile	Perceive multi-channel and multivariable feedback
	[89]	Zhang, T. (2023)	Tactile	A sensorimotor-inspired grasping strategy for a dexterous prosthetic hand is proposed to improve grasping performance
	[65]	Yanchao Wang (2022)	Current/position	Linear judgment analysis algorithm realizes dual closed-loop control
	[33]	Marco Controzzi (2017)	Vibration/tactile	A single actuator realizes the abduction/retraction of the thumb and the flexion/extension of the index finger.
	[90]	Kanaris, I. (2016)	Audio	Cross-modal audio feedback structure
	[48]	Zhou, Hao (2022)	Position/tactile	Based on mode recognition
	[49]	Yoshiko Yabuki (2019)	Pressure	Super elastic makeup glove
	[91]	Leone, F. (2023)	Force	The simultaneous real-time control of hand/wrist gestures and force levels
	[92]	Xu, H. P. (2023)	Tactile	Flexible piezoresistive tactile sensor arrays are proposed for enhancing the sensing intelligence of the untethered soft prosthetic hand
visual	[93]	Pascal Weiner (2018)	Position	Integrating proprioception sensor information and visual information
	[94]	Uikyum Kim (2021)	Tactile	Connect to the robot arm, and at the same time adapt to various favorable features

**Table 4:** Comparative analysis of prosthetic hand control techniques and their efficacies.

Researcher(s)	Year	Methodology	Classifier(s) Used	Accuracy	Note
R Zhang et al. [117,118]	2022	Statistical analysis of sEMG	K-NN, SVM, ANN	94–98.4%	Gender-specific algorithms applied
X Li et.al [119]	2022	Force myography-based recognition	linear discriminant analysis (LDA), K-nearest neighbor (KNN), artificial neural network (ANN), and support vector machine (SVM)	>90%	Robust against up to 50% noise
M Coskun et.al [120]	2021	1D convolutional neural networks	1D-CNN	94.94%	Recognized six hand gestures
Z Zhang et.al [121]	2020	Time-domain feature extraction and ANN	ANN	91.10%	Optimal recognition with seven channels
A Altameem et.al [122]	2022	Feature extraction from EEG (like FFT, continuous wave transform (CWT))	XG Boost	88%	Focused on amputee hand control
G Pan et.al [123]	2018	Decoding gestures from ECoG with RNNs	RNN	90%	Decoded three gestures
G Ghazaei et.al [124]	2017	Deep learning for object classification	CNN	80%	Improved object manipulation

## Data Availability

The data used to support the findings of this study are available from the corresponding author upon request.
